# Combination therapy of ofatumumab and daratumumab in patients with severe anti-NMDA receptor encephalitis

**DOI:** 10.3389/fimmu.2025.1681884

**Published:** 2025-10-27

**Authors:** Baojie Wang, Yufeng Chu, Chao Zhang, Shougang Guo

**Affiliations:** ^1^ Department of Neurology, Shandong Provincial Hospital Affiliated to Shandong First Medical University, Jinan, China; ^2^ Department of Neurology, Tianjin Medical University General Hospital, Tianjin, China

**Keywords:** ofatumumab, daratumumab, N-methyl-D-aspartate receptor (NMDAR) antibody encephalitis, immunotherapy, prognosis

## Abstract

N-methyl-D-aspartate receptor (NMDAR) antibody encephalitis (NMDARE) is an autoimmune disorder in which approximately 25% of patients develop refractory disease with severe prolonged neurological deficits. Currently, there is no consensus on optimal treatment for NMDARE refractory to first- or second-line therapies. We present two cases of severe refractory NMDARE treated with novel combination therapy of ofatumumab and daratumumab. We describe two adolescent female patients with severe neuropsychiatric manifestations unresponsive to standard first-line therapies, including intravenous methylprednisolone (IVMP) and intravenous immunoglobulins (IVIG). Following 5–6 cycles of ofatumumab and daratumumab combination therapy, both patients achieved significant functional improvement (modified Rankin Scale [mRS] score ≤ 2) within 12 weeks. Serological analysis revealed undetectable NMDAR-IgG levels and rapid depletion of circulating CD19^+^ B cells and CD38^+^ antibody-secreting cells (ASCs) within 2 weeks. Treatment-related adverse events were acceptable. This case report demonstrates that combined treatment of ofatumumab and daratumumab therapy is well-tolerated and may represent a novel therapeutic strategy for severe NMDARE. The observed rapid serological and clinical responses highlight its potential efficacy, warranting further investigation in large cohorts.

## Introduction

Anti-N-methyl-D-aspartate receptor (NMDAR) antibody encephalitis (NMDARE) is an autoimmune disease characterized by autoantibody production, typically presenting with a spectrum of neuro-psychiatric manifestations (1). Within the established treatment landscape, defined as first-line immunotherapy followed by second-line treatment such as rituximab, NMDARE can be controlled in most patients. However, approximately 25% of patients develop refractory with persistent severe deficits despite these interventions, and no consensus exists for managing refractory cases _(_ 2, 3_)._

Pathogenesis of NMDARE involves autoantigen-specific B cells in secondary lymphoid tissues and auto antibody-secreting plasma cells (ASCs), which produce pathogenic antibodies targeting the extracellular domain of the NMDAR NR1 subunit (4). Notably, unlike short-lived plasmablasts, non-expanding long-lived plasma cells reside in specialized survival niches within the bone marrow or inflamed tissues to sustain pathogenic autoantibody production and escape from conventional therapies such as rituximab, which fails to eliminate differentiated plasma cells (5). This mechanistic insight has facilitated the rational development of mechanism-targeted therapies. Among emerging treatments, daratumumab–a human IgGκ monoclonal antibody targeting CD38, holds promise by suppressing CD38^+^ plasma cells and subsequent autoantibody secretion. Additionally, as CD38 is expressed on activated T cells, daratumumab may exert immunomodulatory effects by increasing T-cell clonality and attenuating the immune suppressive activity of CD38+ regulatory T cells (6).

Daratumumab is currently approved for multiple myeloma, either as monotherapy or in combination regimens. Preliminary studies provide considerable clinical benefit in patients with lupus and immune thrombocytopenia (7, 8), as well as in neurologic antibody-mediated diseases (9), with manageable safety profiles. We report the first dual-target therapy combining anti-CD20 monoclonal antibody (mAb) ofatumumab and anti-CD38 mAb daratumumab in two cases of severe NMDARE patients. As a result, both patients achieved functional independence (modified Rankin Scale score ≤2) within 12 weeks, supporting this strategy’s therapeutic potential.

## Case presentations

Informed consents were signed by the participants or the legal representatives.

### Case 1

A 15-year-old girl was referred from an external hospital with a confirmed diagnosis of NMDARE. On admission, she presented with status epilepticus, involuntary movements of the orofacial and left upper limb, and coma despite unremarkable vital signs, requiring intensive care unit (ICU) and mechanical ventilation (modified Rankin scale, mRS 5; Clinical Assessment Scale in Autoimmune Encephalitis, CASE 26; Glasgow coma scale, GCS 3). Her symptoms had begun 4 weeks prior with abnormal psychiatric behaviors. Lumbar puncture (LP) in the external hospital revealed pleocytosis with normal protein and glucose level in cerebrospinal fluid (CSF). NMDAR-IgG antibodies were detected in serum (1:1000) and CSF (1:100) by cell-based assay.

**Figure 1:**
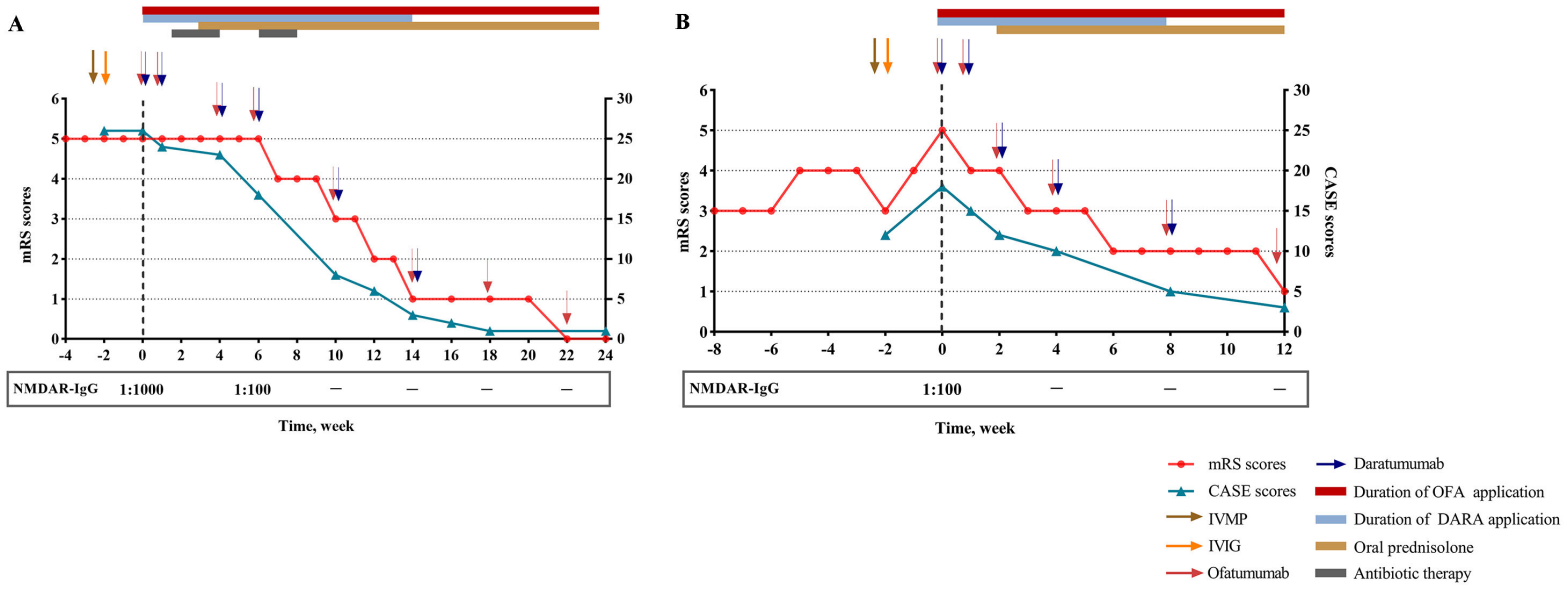
Clinical course of patient 1 **(A)** and patient 2 **(B)**. NMDAR, N-methyl-D-aspartate receptor; mRS, modified Rankin scale; CASE, Clinical Assessment Scale in Autoimmune Encephalitis; IVIG, intravenous immunoglobulin; IVMP, intravenous methylprednisolone; OFA, ofatumumab; DARA, daratumumab.

Despite standard first-line treatment (intravenous methylprednisolone, IVMP: 1 g/d × 5; intravenous immunoglobulin, IVIG: 0.4g/kg/d × 5), followed by slow prednisone tapering, she remained comatose with generalized convulsive seizures and absent withdrawal to noxious stimulation. Owing to the aggressive course of the disease, combined immunotherapy with ofatumumab (20 mg/week subcutaneously) and daratumumab (8 mg/kg/week intravenously) was initiated at week 0 (**Figure 1A**). After two courses of combined therapy (week 0, 1), her episodes of seizure ceased (mRS 5; CASE 23) and GCS improved to 6. Post-treatment flow cytometry after the second cycle confirmed complete depletion of CD19^+^ B cells and CD38^+^ ASCs. However, on day 17, the patient developed fever (38.°C) secondary to bloodstream infections. Repeated chest CT demonstrated bilateral hypostatic pneumonia. Bacterial culture of bronchoalveolar lavage fluid (BALF) obtained via bronchoscopy revealed Pseudomonas aeruginosa. Blood culture was positive for Klebsiella pneumoniae. Immunotherapy was temporarily suspended, and meropenem was added. After three weeks of intensive antimicrobial therapy and management of complications, infection markers (procalcitonin, C-reactive protein (CRP), white blood cell counts) normalized. Thus, the combined immunotherapy was subsequently resumed to deal with residual critical neurological deficits (mRS 5; CASE 18; GCS 11). Over the next three cycles of combined immunotherapy (week 4, 6, 10), she achieved rapid symptom resolution, regained full consciousness, and followed simple commands. She was transferred from ICU to rehabilitation at week 10 (mRS 3; CASE 8; GCS 15). By 14 weeks post-treatment initiation, neurological examination was normal except for mild memory/cognitive impairment (Mini-Mental State Examination, MMSE 25; Montreal Cognitive Assessment, MoCA: 26). Daratumumab was discontinued, while ofatumumab every 4 weeks continues as maintenance therapy. At 8 months follow-up, the patient has been stable with normal cognition and returned to school.

### Case 2

A 17-year-old girl suddenly developed persecutory delusion, restlessness, and incoherent speech 8 weeks prior to admission. On first admission, she received treatment for schizophrenia for over one month in the department of psychiatry at external hospital without clinical improvement. Subsequently she was referred to neurology services for comprehensive investigations including serologic and CSF investigations as well as MRI of brain and electroencephalogram (EEG). Brain MRI showed frontal lobe of T2/FLAIR hyperintensity (**Supplementary Figure 1**). The EEG revealed diffuse activity (1.5–3 Hz) with delta brush. CSF revealed elevated protein concentration with normal glucose, chloride and leukocyte count. No infectious etiology could be demonstrated. The serum-CSF pair was sent for antibody testing, with positive for NMDAR-IgG antibodies (serum titer 1:100; CSF titer 1:32), establishing a definitive diagnosis of NDARE. First-line immunotherapy was initiated with IVMP (1 g/d for 5 days) and IVIG (0.4 g/kg/d for 5 days), with prednisone oral tapering.

However, the patient deteriorated rapidly, worsening of psychiatric deficit and cognitive impairment, together with evolving new-onset generalized tonic-clonic seizure and involuntary movement of left upper limb (mRS 5; CASE 18; GCS 6). In the absence of clinical improvement to first-line treatment, she was administrated with combined therapy comprising ofatumumab 20 mg loading doses subcutaneously and daratumumab 8 mg/kg intravenously at week 0, 1, 2 and every 4 weeks thereafter. Upon four cycles combined treatment (week 0, 1, 2, 4), she improved promptly with a complete resolution of seizure. Functionally, the patient regained her ability to walk without support (mRS 3; CASE 10; GCS 13). However, she had ongoing severe cognitive dysfunction with disorientation in time, impaired language and executive functions, and below-average scores on processing speed, attention, perception and construction (MMSE 10; MoCA 4). After completion of 5 sessions of combined treatment at 8 weeks, symptoms improved rapidly as demonstrated by a reduction of the CASE score from 18 at the initiation of combined treatment to 5. Concurrently, the patient showed a slight increase in cognitive functions (MMSE 18; MoCA 13). With sustained clinical recovery (mRS 2; CASE 5; GCS 15), daratumumab was discontinued. The patient continues to receive ofatumumab monthly. Infusions/injection were well tolerated, and the patient did not develop adverse events related to combined therapy (**Figure 1B**).

**Figure 2:**
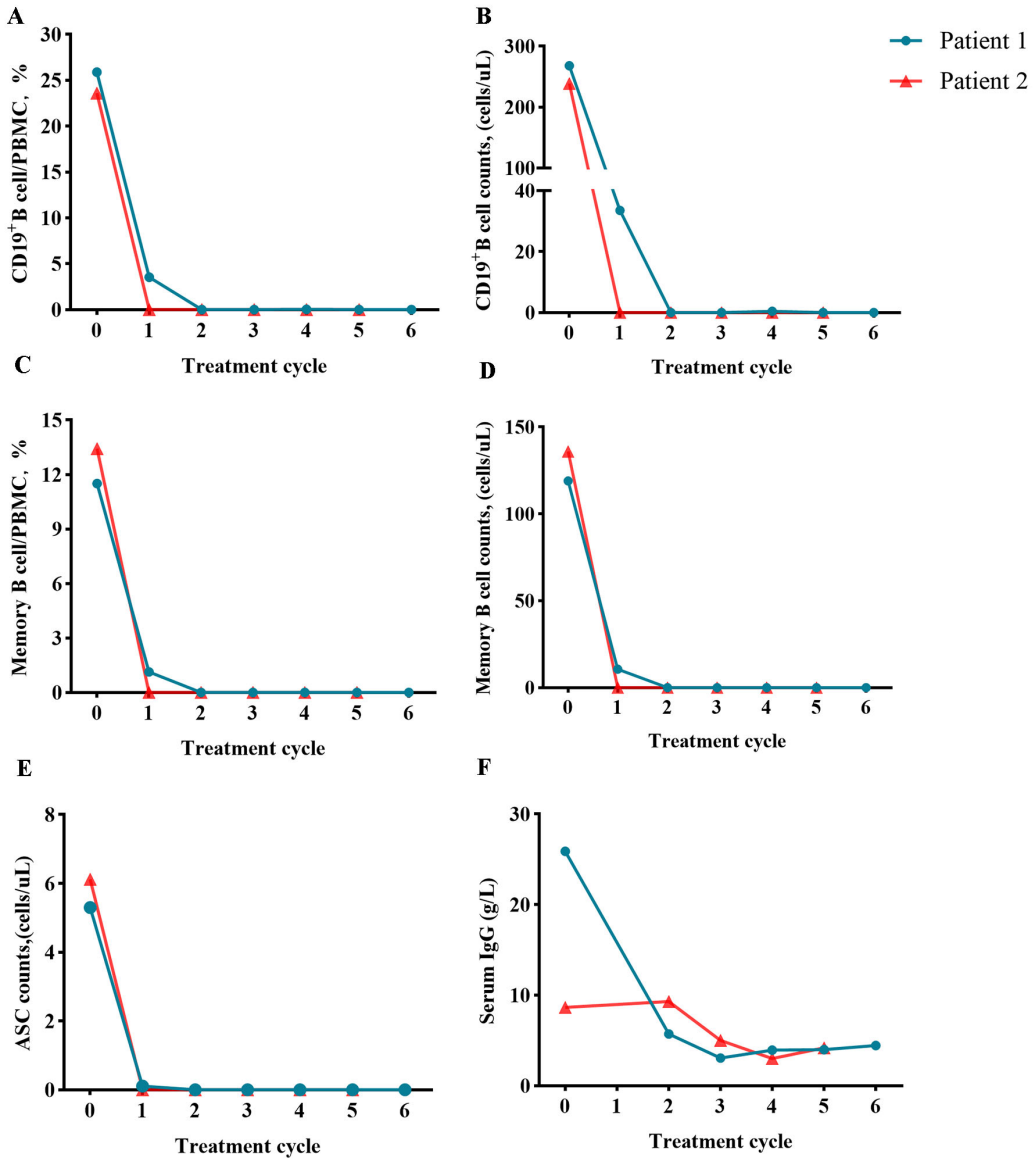
Assessment of B-cell population before and after ofatumumab and daratumumab treatment (day 0). Changes in the proportion or number of CD19^+^ B cells **(A, B)**, memory B cells **(C, D)**, ASCs **(E)** and serum IgG concentration **(F)**.

Detailed caracteristics of patients,B-Cell Population, ASCS, IgG concentration before and after combined treatment are shown in **Table 1** and **Figures 2A–F**.

**Table 1:** Characteristics of patients with severe NMDARE receiving combined therapy.

Characteristic	Patient 1	Patient 2
Age at disease onset, years	15	17
Gender	Female	Female
Clinical symptoms	Status epilepticus, consciousness disturbance, involuntary movement	Psychiatric symptoms, seizures, involuntary movement, cognitive impairment
Auxiliary examination		
Serum Anti-NMADR antibody titer	1:1000	1:100
CSF Anti-NMDAR antibody titer	1:100	1:32
Leukocyte count in CSF (106/L)	15	8
Protein concentration in CSF (mg/L)	304	600
Oligoclonal bands	Negative	Negative
MRI	Normal	Bilateral frontal lobe lesions
EEG	Discontinuous epileptic discharges	Diffuse slowing, delta brush
Peripheral CD19+ B cells/PBMCs, %	25.9	23.59
Peripheral CD19+ B cells, counts/μL	268	239
Peripheral CD27+ Memory B cells/PBMCs, %	11.51	13.43
Peripheral CD27+ Memory B cells, counts/μL	119	136
Peripheral CD38+ ASCs, counts/μL	5	6
Baseline score		
NEOS scores	3	2
mRS scores	5	5
CASE scores	26	18
GCS scores	3	6
Admission to the ICU	Yes	Yes
Mechanical ventilation	Yes	No
Teratoma	None	None
First-line immunotherapy	IVMP+IVIG	IVMP+IVIG
Latency symptom onset to first-line treatment initiation, days	24	44
Duration of disease at combined treatment, days	38	59
Follow up		
Neurological outcomes at 4 weeks	mRS:5; CASE:23; GCS:11	mRS:3; CASE:10; GCS:13
Neurological outcomes at 12 weeks	mRS:2; CASE:6; GCS:15	mRS:1; CASE:3; GCS:15
OFA cycles	9	6
DARA cycles	6	5
Treatment-related adverse events	Upper respiratory infection	None
Time of follow-up (since DARA initiation), months	7	4

NMDAR, N-methyl-D-aspartate receptor; CSF, cerebrospinal fluid; EEG, electroencephalogram; MRI, magnetic resonance imaging; ASC, antibody-secreting plasma cells; NEOS, the NMDAR Encephalitis One-Year Functional Status; mRS, modified Rankin scale; CASE, Clinical Assessment Scale in Autoimmune Encephalitis; GCS, Glasgow coma scale; ICU, intensive care unit; IVIG, intravenous immunoglobulin; IVMP, intravenous methylprednisolone; OFA, ofatumumab; DARA, daratumumab.

## Discussion

In these two cases of severe NMDARE refractory to first-line immunotherapy, combined application of ofatumumab and daratumumab induced rapid depletion of circulating CD19^+^ B cells and CD38^+^ ASCs within 2 weeks, accompanied by serological clearance of NMDAR-IgG antibodies. This suggests that dual targeting of B-cell precursors and long-lived plasma cells may overcome therapeutic resistance.

In ICU-dependent NMDARE, first-line treatment alone is associated with limited clinical improvement. Early initiation of second-line or escalated immunotherapy appears to be a promising strategy to accelerate recovery and reduce the duration of ICU stay. Among second-line immunotherapies, the use of rituximab increased from 13.5% to 28.3%, with a more pronounced rise in the subgroup of patients with severe disease (mRS score of 5) at initial presentation (10). This agent may offer a faster onset of action compared to azathioprine and mycophenolate mofetil (MMF). Our decision to administer ofatumumab was based on pathological study demonstrating that germinal center hyperactivity in lymphoid tissue drives ongoing NR1-IgG production by autoreactive B cells (11). Subcutaneous administration of ofatumumab enables transit from the hypodermis to the lymphatic system prior to entering systemic circulation, potentially leading to more profound depletion of immune cells within lymph node (12). Furthermore, ofatumumab demonstrates greater antibody-dependent cellular cytotoxicity(ADCC) activity and higher complement-dependent cytotoxicity(CDCC) activity than rituximab (13).

In our study, daratumumab was associated with a significant reduction in pathogenic NMDAR-IgG antibodies in these two patients, that suggest an effective depletion of long-lived plasma cells. The fact that in patients refractory to previous immunosuppressive and B-cell depleting therapies further emphasizes the therapeutic relevance of targeting long-lived plasma cells in NMDARE, as noted previously (2, 3). In refractory autoimmune encephalitis, long-lived plasma cells in meningeal compartments, lymph node sinusoids and parenchymal are responsible for pathogenic antibody production (14, 15). Although rituximab therapy included B-cell depletion, an increase in co-expression of CD38 and HLA-DR on both CD4^+^ and CD8^+^ memory T cells has been observed at baseline (9). Additionally, rituximab may induce a compensatory shift from autoantibody-producing B cells to plasma cells. The inflammatory milieu resulting from B cell depletion further promotes the differentiation and settlement of long-lived plasma cells in the spleen, identifying these cells as a relevant therapeutic target (16).

The combination of ofatumumab and daratumumab may deplete peripheral B cells and ASCs, potentially reducing antibody trafficking and infiltration of autoreactive B cells and plasma cells into the brain. In treatment-refractory antibody-mediated neurological disorders, daratumumab treatment has been associated with reductions in CD38-expressing T cells, NK cells, and serum neurofilament light chain levels, suggesting immunomodulatory effects and attenuation of active axonal injury (9). In NK/T-cell lymphoma (17), daratumumab can cross the blood-brain barrier, highlighting its potential in CNS involvement where antibody-secreting cells are found in perivascular, interstitial, and Virchow-Robin spaces, and where B and T cells are mainly located in perivascular regions (15).

In our study, both patients achieved significant functional improvement (mRS ≤ 2) in our study. This aligns with a retrospective evidence showing plasma cell-depleting therapy enables refractory autoimmune encephalitis patients to attain outcomes comparable to patients already responding to standard first- and/or second-line therapies (2). Notably, these two patients treated with concomitant ofatumumab and daratumumab initiation showed a rapid decrease in CASE score and mRS score within 12 weeks post-treatment, contrasting with prolonged response times in previous studies, where daratumumab was typically initiated as an escalation therapy when patients unresponsive to a multimodal immunotherapy (2, 9, 18, 19). This finding also underscores the critical need for prompt initiation of combined high-efficacy immunotherapy in severe NMDARE patients without active infection, to facilitate clinical improvement and prevent permanent neurologic dysfunction.

In this study, both patients demonstrated favorable tolerance with manageable treatment-related adverse events. This favorable tolerability is likely attributable to the use of reduced-dose daratumumab (50% of standard) and early discontinuation upon patients achieving mRS score ≤2. Although IgG levels decreased over time in both patients, follow-up to date has shown no associated increase in infection risk. Previous studies for refractory lupus nephritis, immune thrombocytopenia and multiple myeloma reported a reasonable safety profile of daratumumab (7, 8, 20). Infusion-related reactions occurred in 35% -71% and were of grade 1 or 2. Infusion rate may be important for the management of infusion-related reactions (20). Most of the adverse events observed with daratumumab were of grade 1 or 2. Grade 3 or 4 adverse events were reported in 26%-53% of the patients including pneumonia, urinary tract and blood stream infection, neutropenia, leukopenia, anemia, and hyperglycemia. The most frequent serious adverse events were infection-related events, which occurred in 10%-17% of the patients (8, 20). For critically NMDARE patients in ICU who are already on mechanical ventilation, the risk of respiratory tract infection may be correspondingly elevated. Therefore, a comprehensive evaluation patient’s age, airway management, history of immunosuppressant use, as well as B-cell, plasma cell, and immunoglobulin levels should be conducted before determining the appropriate timing for pharmacological intervention.

Our study has several limitations. First, as a case report including only two patients, the generalizability of our findings is inherently limited, and these results must be confirmed in larger, controlled cohorts. Second, although the follow-up period demonstrated sustained improvement, it remains relatively short; long-term data are needed to confirm the overall safety profile in the setting of NMDARE.

In conclusion, for refractory NMDARE requiring prolonged ICU care, this dual-target strategy represents a promising rescue therapy. Future studies are needed to address complex interplay between immune cells and the immune microenvironment, that is predictive of clinical outcome and depth of treatment response in severe NMDARE patients treated with highly effective combinations treatment containing ofatumumab and daratumumab.

## Data Availability

The original contributions presented in the study are included in the article/Supplementary Material. Further inquiries can be directed to the corresponding authors.
