# Supplementary figures and images for: Combination therapy of ofatumumab and daratumumab in patients with severe anti-NMDA receptor encephalitis
Source: Front Immunol. 2025 Oct 27;16:1681884. doi: 10.3389/fimmu.2025.1681884 (PMC12597730; doi:10.3389/fimmu.2025.1681884)

**eFigure 1 Brain MRI For Patient 2**


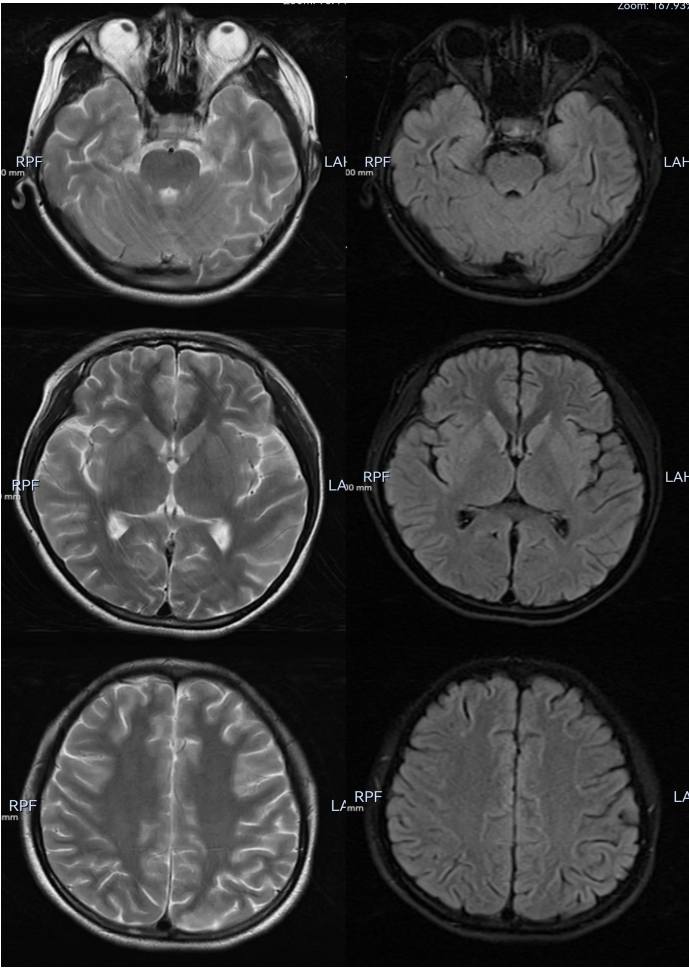


Brain MRI showed frontal lobe of T2/FLAIR hyperintensity.

Supplement: Supplementary file 1 [file DataSheet1.docx]
